# Disruption of Iron Homeostasis and Mitochondrial Metabolism Are Promising Targets to Inhibit Candida auris

**DOI:** 10.1128/spectrum.00100-22

**Published:** 2022-04-12

**Authors:** Claudia Simm, Harshini Weerasinghe, David R. Thomas, Paul F. Harrison, Hayley J. Newton, Traude H. Beilharz, Ana Traven

**Affiliations:** a Infection Program and the Department of Biochemistry and Molecular Biology, Biomedicine Discovery Institute, Monash University, Victoria, Australia; b Centre to Impact AMR, Monash University, Victoria, Australia; c Department of Microbiology and Immunology, University of Melbourne at the Peter Doherty Institute for Infection and Immunity, Melbourne, Australia; d Bioinformatics Platform, Monash University, Victoria, Australia; e Development and Stem Cells Program and the Department of Biochemistry and Molecular Biology, Biomedicine Discovery Institute, Monash University, Victoria, Australia; Septomics Research Center, Friedrich Schiller University and Leibniz Institute for Natural Product Research and Infection Biology - Hans Knöll Institute

**Keywords:** *Candida auris*, antifungal agents, fungal pathogens, iron, metabolism, mitochondrial metabolism

## Abstract

Fungal infections are a global threat, but treatments are limited due to a paucity in antifungal drug targets and the emergence of drug-resistant fungi such as Candida auris. Metabolic adaptations enable microbial growth in nutrient-scarce host niches, and they further control immune responses to pathogens, thereby offering opportunities for therapeutic targeting. Because it is a relatively new pathogen, little is known about the metabolic requirements for C. auris growth and its adaptations to counter host defenses. Here, we establish that triggering metabolic dysfunction is a promising strategy against C. auris. Treatment with pyrvinium pamoate (PP) induced metabolic reprogramming and mitochondrial dysfunction evident in disrupted mitochondrial morphology and reduced tricarboxylic acid (TCA) cycle enzyme activity. PP also induced changes consistent with disrupted iron homeostasis. Nutrient supplementation experiments support the proposition that PP-induced metabolic dysfunction is driven by disrupted iron homeostasis, which compromises carbon and lipid metabolism and mitochondria. PP inhibited C. auris replication in macrophages, which is a relevant host niche for this yeast pathogen. We propose that PP causes a multipronged metabolic hit to C. auris: it restricts the micronutrient iron to potentiate nutritional immunity imposed by immune cells, and it further causes metabolic dysfunction that compromises the utilization of macronutrients, thereby curbing the metabolic plasticity needed for growth in host environments. Our study offers a new avenue for therapeutic development against drug-resistant C. auris, shows how complex metabolic dysfunction can be caused by a single compound triggering antifungal inhibition, and provides insights into the metabolic needs of C. auris in immune cell environments.

**IMPORTANCE** Over the last decade, Candida auris has emerged as a human pathogen around the world causing life-threatening infections with wide-spread antifungal drug resistance, including pandrug resistance in some cases. In this study, we addressed the mechanism of action of the antiparasitic drug pyrvinium pamoate against C. auris and show how metabolism could be inhibited to curb C. auris proliferation. We show that pyrvinium pamoate triggers sweeping metabolic and mitochondrial changes and disrupts iron homeostasis. PP-induced metabolic dysfunction compromises the utilization of both micro- and macronutrients by C. auris and reduces its growth *in vitro* and in immune phagocytes. Our findings provide insights into the metabolic requirements for C. auris growth and define the mechanisms of action of pyrvinium pamoate against C. auris, demonstrating how this compound works by inhibiting the metabolic flexibility of the pathogen. As such, our study characterizes credible avenues for new antifungal approaches against C. auris.

## INTRODUCTION

Life-threatening infections with fungi have become more prevalent over the last 20 to 30 years, now killing more than 1.5 million people annually (1). Despite this increasing threat, we have very few antimycotic drugs and our antifungal drug arsenal has not changed much in the last 30 years (2). The problem has been exacerbated by the emergence of Candida auris, which presents an urgent health challenge and has been identified as a serious global threat to humans (3). This yeast emerged as a human pathogen nearly simultaneously in different geographical locations subdivided into four genetically distinct clades, with a potential fifth one discovered recently (4, 5). C. auris is a nosocomial pathogen with a mortality rate of 30% to 70% (6–8). In contrast to other *Candida* species, this fungus is a strong colonizer of the human skin, which facilitates transmissions between patients (9, 10). This trait, coupled with its environmental persistence on hospital surfaces and medical equipment, has caused hospital outbreaks, including as a coinfection of seriously ill COVID-19 patients (11–13). Most C. auris isolates are resistant to at least one of the three main classes of antifungal drugs (azoles, echinocandins, and polyenes) with 86 to 93%, 8 to 35%, and 2 to 7% no longer susceptible to fluconazole, amphotericin B, and caspofungin, respectively (10). Alarmingly, there have been reports of panresistant clinical isolates (4, 14, 15). Given that C. auris is resistant to all available antifungals coupled with limited target space (either membrane or cell wall integrity), improved strategies to combat this multidrug-resistant pathogen rely on characterizing new pathways for therapeutic intervention.

We are interested in exploring metabolism as a target for antifungal strategies. Manipulation of metabolism is promising for several human diseases, such as cancer, inflammation, and autoimmunity (16–18), but is less understood in infectious diseases. There is good evidence that metabolism might provide the much-needed expanded target space to search for new antifungal therapies. Indeed, an intricate cross talk between pathogen and host metabolic pathways regulates fungal growth, immune responses, and ultimately host survival of infection (19, 20). Fungal pathogens need metabolic adaptability because host niches can be scarce in both macronutrients, such as carbon sources, and micronutrients, such as metals (19, 21, 22). Moreover, hosts can actively restrict nutrients for pathogens. A key example of this is the battle for iron (23, 24).

Host organisms actively sequester or compartmentalize iron to drastically reduce its uptake by invading fungal pathogens, a defense mechanism termed nutritional immunity (25). Upon infection with microorganisms, macrophages increase the expression of the natural resistance-associated protein 1 (NRAMP1), a metal transporter that actively pumps iron and manganese ions from the phagolysosome (where microbes reside) into the cytoplasm, thus restricting access to these metals (26). Macrophages are actively recruited to fungal lesion sides to restrict access to iron (27). Moreover, access to iron is further restricted by an increased expression of hepcidin blocking the release of this metal from macrophages (23). In turn, fungal pathogens have developed strategies to overcome the low bioavailability of iron by expressing high-affinity iron uptake and scavenging proteins. For example, Candida albicans uses three uptake systems to acquire iron (28, 29): the reductive iron uptake by ferric reductases (encoded by *FRE*/*CFL* genes) for the reduction of Fe^3+^ to Fe^2+^, followed by the oxidation of ferrous iron by the ferroxidase Fet3, and lastly ferric iron import by the permease Ftr1. C. albicans further utilizes xenosiderophores for iron import via the siderophore transporter Sit1 (30, 31) and can scavenge hemoglobin or hemin from its host followed by the release of iron through the heme oxygenase Hmx1 (32).

Most of what we know about metabolism in fungal infections comes from studies of a few exemplar pathogens, such as C. albicans, Aspergillus fumigatus, and Cryptococcus neoformans. While it can be expected that some general principles will hold across fungal species, differences have been reported in their metabolic behaviors. For example, while both C. albicans and A. fumigatus induce aerobic glycolysis in macrophages, only C. albicans hijacks this to deplete glucose and kill macrophages (33, 34). It has also been shown that C. neoformans is less metabolically flexible than C. albicans following treatment with inhibitors of mitochondrial respiration (35). As a relatively new human pathogen, the metabolic requirements for growth and immune interactions of C. auris are largely unknown.

Here, we report that inducing metabolic dysfunction is a promising strategy against C. auris to repress fungal proliferation and enhance antifungal drug susceptibility. Our mechanistic studies showed that the anthelmintic drug pyrvinium pamoate (PP) inhibits growth of C. auris through multipronged metabolic repression, disrupting the ability of the pathogen to utilize both micro- and macronutrients. We discovered that PP enhances nutritional immunity and disrupts iron homeostasis in C. auris, which in turn causes wide-spread alteration of carbon and lipid metabolism, lower activity of the TCA cycle enzyme aconitase, and mitochondrial dysfunction. These effects are likely due to the requirement for iron-sulfur clusters and heme as cofactors for metabolic enzymes and the mitochondrial respiratory chain. Indeed, a highly expanded set of the siderophore acquisition genes in C. auris relative to that in C. albicans (36) suggested to us that its iron needs might be an Achilles heel, which our data fully support. PP inhibited the proliferation of C. auris in macrophages without majorly affecting the viability or metabolism of host cells at antifungal concentrations, showing its efficacy and selectivity in an immune infection system. Collectively, our findings explain the antifungal mechanism of action of PP and establish metabolism, mitochondria, and iron homeostasis as credible targets for the development of improved antifungal strategies against C. auris. We further shed light on the metabolic needs of C. auris for its extensive proliferation inside immune phagocytes and demonstrate how fungal metabolic plasticity could be inhibited pharmacologically to reduce pathogenic loads.

## RESULTS

### Pyrvinium pamoate inhibits growth of C. auris.

**FIG 1:**
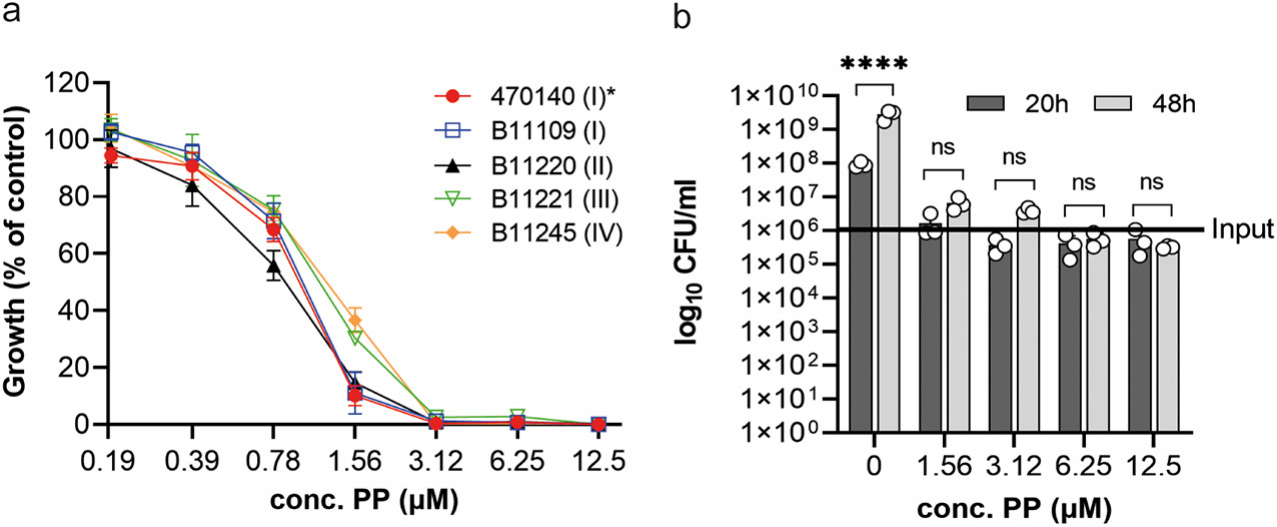
Pyrvinium pamoate inhibits the growth of C. auris. (a) C. auris isolates were grown in RPMI 1640 medium according to CLSI-M27 procedure, and cell density at 600 nm was measured after 20 h of growth at 37°C. The clades of the isolates are indicated in brackets. *, the C. auris isolate 470140 was used for all subsequent experiments. The data shown are the means ± standard error of mean (SEM; *n* = 4). (b) C. auris was inoculated at a cell density of 0.1 and treated with PP. After 20 h and 48 h of incubation at 37°C, cell cultures were diluted and aliquots were plated onto YPD plates. CFU were counted after 2 days of incubation at 30°C. Error bars indicate SEM (*n* = 3). Each 20-h time point was compared to its corresponding 48-h time point with a one-way analysis of variance (ANOVA) with Šídák’s multiple-comparison test (****, *P* < 0.0001).

To identify compounds with new activities against C. auris, we determined the efficacy of pyrvinium pamoate (PP). PP is an oral FDA-approved drug for parasitic worm infections that is well tolerated in humans, albeit showing very little systemic absorption (37). We performed MIC assays as per CLSI guidelines, testing strains belonging to C. auris clades I to IV. All of the tested isolates were susceptible toward PP with an MIC of 3.1 μM (Fig. 1a and Table 1). Screens of the off-patent Prestwick Chemical library (which contains PP as one of the compounds) support our results of PP’s antifungal activity against C. auris (38–40). We did not observe interclade variability in susceptibility, hinting at a target not associated with genetic divergence between the clades. Similarly, we found little difference in the 50% inhibitory concentration (IC_50_) values between the tested isolates (Table 1).

**TABLE 1:** MIC and IC_50_ values of pyrvinium pamoate against C. auris isolates

Name	Clade	MIC (μM/μg/mL)	IC_50_ (μM/μg/mL)
470140	I	3.1/1.78	1.0/0.57
470121	I	3.1/1.78	1.1/0.63
B11098	I	3.1/1.78	1.4/0.81
B11109	I	3.1/1.78	1.0/0.57
B11203	I	3.1/1.78	1.6/0.92
B11205	I	3.1/1.78	0.9/0.52
B8441	I	3.1/1.78	1.4/0.81
B11220	II	3.1/1.78	0.9/0.52
B11221	III	3.1/1.78	1.1/0.63
B11222	III	3.1/1.78	1.1/0.63
B11244	IV	3.1/1.78	1.2/0.69
B11245	IV	3.1/1.78	1.2/0.69

Next, we investigated if PP inhibits cell growth (fungistatic) or kills C. auris cells (fungicidal) by performing minimal fungicidal concentration (MFC) experiments. C. auris cultures were grown in presence or absence of PP for 20 and 48 h, after which an aliquot of the cultures was removed, diluted, and plated onto yeast extract-peptone-dextrose (YPD) plates. After 2 days of incubation at 30°C, CFU were counted. We observed a 2-fold increase in cell number compared to the initial input of C. auris cells at the start of the assay for 1.56 μM PP (1/2× MIC) at 20 h, which increased to 6-fold at 48 h. Approximately 5 × 10^5^ cells were counted for 1× MIC, 2× MIC, and 4× MIC at 20 h, which is half the original input, with no further decrease in a concentration-dependent manner (Fig. 1b). The number of survivor cells did not significantly change at 48 h compared to their corresponding values at 20 h, suggesting a lack of a time-dependent fungicidal action. Since the MFC is defined as the concentration where growth of the initial inoculum is reduced by 99.9% (41, 42), none of the tested concentrations of PP at any time point had achieved the MFC, indicating that PP inhibits C. auris growth in a fungistatic but not fungicidal manner.

### Pyrvinium pamoate induces metabolic reprogramming in C. auris.

To gain insight into the mechanism of action of PP against C. auris, we performed transcriptome analysis and compared cultures with or without drug. To determine optimal conditions for this analysis, we performed a short-term treatment CFU assay. The addition of 5 μM PP at any time point revealed a reduction of viable cells between 60% to 80% (Fig. S1). While this seems to contradict previously described MFC results where cell numbers were reduced by only 50% at a similar concentration after 20 and 48 h, this phenomenon has been documented before (41, 43). For instance, cell counts of C. albicans treated with amphotericin B rapidly declined within 2 h of treatment but increased again 12 h after treatment, approaching near control values (42). Treatment of C. auris cultures with 2 μM PP showed a 50% reduction in colonies for all time points greater than 15 min. The addition of 1 μM PP for 30 min showed a reduction of growth without displaying much cytotoxicity, thereby reducing the risk of off-target or secondary transcriptional changes due to cell death (Fig. S1). Therefore, this treatment condition was chosen for the RNA-seq experiment. The RNA-seq data can be interactively viewed at https://degust.erc.monash.edu/degust/compare.html?code=3ca3c1202b154234cab0544358026de0#/ and has also been submitted to GEO under accession number GSE176354.

**FIG 2:**
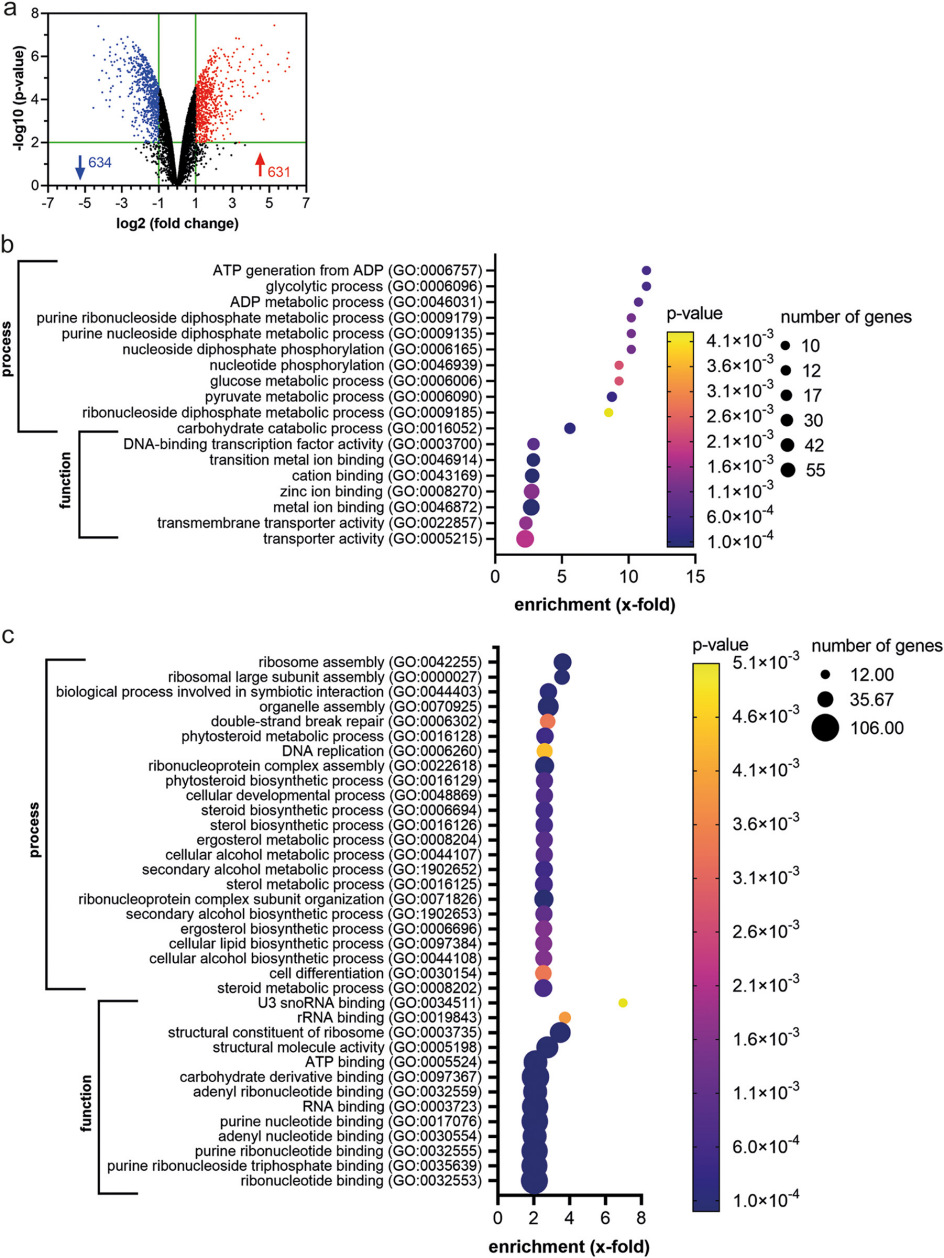
Pyrvinium pamoate induces metabolic reprogramming and downregulation of growth-related functions. (a) Volcano plot showing upregulated (red) and downregulated (blue) genes. Cutoff values are 2-fold up- or downregulated with a *P* value of ≤0.01. Top GO enrichment analysis (PANTHER analysis tool) of (b) upregulated genes and (c) downregulated genes for biological processes and molecular function.

The response of C. auris to PP consisted of 634 down- and 631 upregulated genes (fold change [FC] of ≥2; *P* value of ≤0.01) (Fig. 2a) (Table S2). GO term enrichment analysis was performed with differentially expressed genes (DEGs; PANTHER GO enrichment analysis, Fisher test, Bonferroni correction, *P* value of ≤0.01). For the upregulated genes, 4 out of 11 of the GO enrichment terms in biological processes belong to carbohydrate metabolism (Fig. 2b). Molecular function GO terms include mainly metal binding or transport (Fig. 2b).

Most of the downregulated DEGs associate with cell growth and division, including DNA replication and repair, as well as ribosome biogenesis and translation (Fig. 2c). This is consistent with PP inhibiting C. auris growth. Moreover, there was an enrichment in downregulated genes involved in sterol and ergosterol biosynthesis (Fig. 2c). Detailed KEGG analysis confirmed that all but one gene in the ergosterol biosynthesis pathway showed reduced gene expression in response to PP (Fig. S2a).

The changes in ergosterol biosynthesis genes prompted us to test the effects of PP in combination with current antifungal drugs against C. auris. Fluconazole and amphotericin B exert their antifungal activity by inhibiting ergosterol biosynthesis or availability, while the echinocandin antifungal drug caspofungin targets cell wall biosynthesis by inhibiting 1,3-β glucan synthase. None of these three antifungal compounds showed synergistic interactions with pyrvinium pamoate (Fig. S2b to e). However, fluconazole and to a lesser degree caspofungin were additive with PP (Fig. S2b and c).

Collectively, the RNA-seq data indicate that PP causes large metabolic shifts in C. auris. Below, we further address the specific metabolic effects of PP on C. auris.

### Pyrvinium pamoate reduces TCA cycle activity and causes mitochondrial dysfunction.

**FIG 3:**
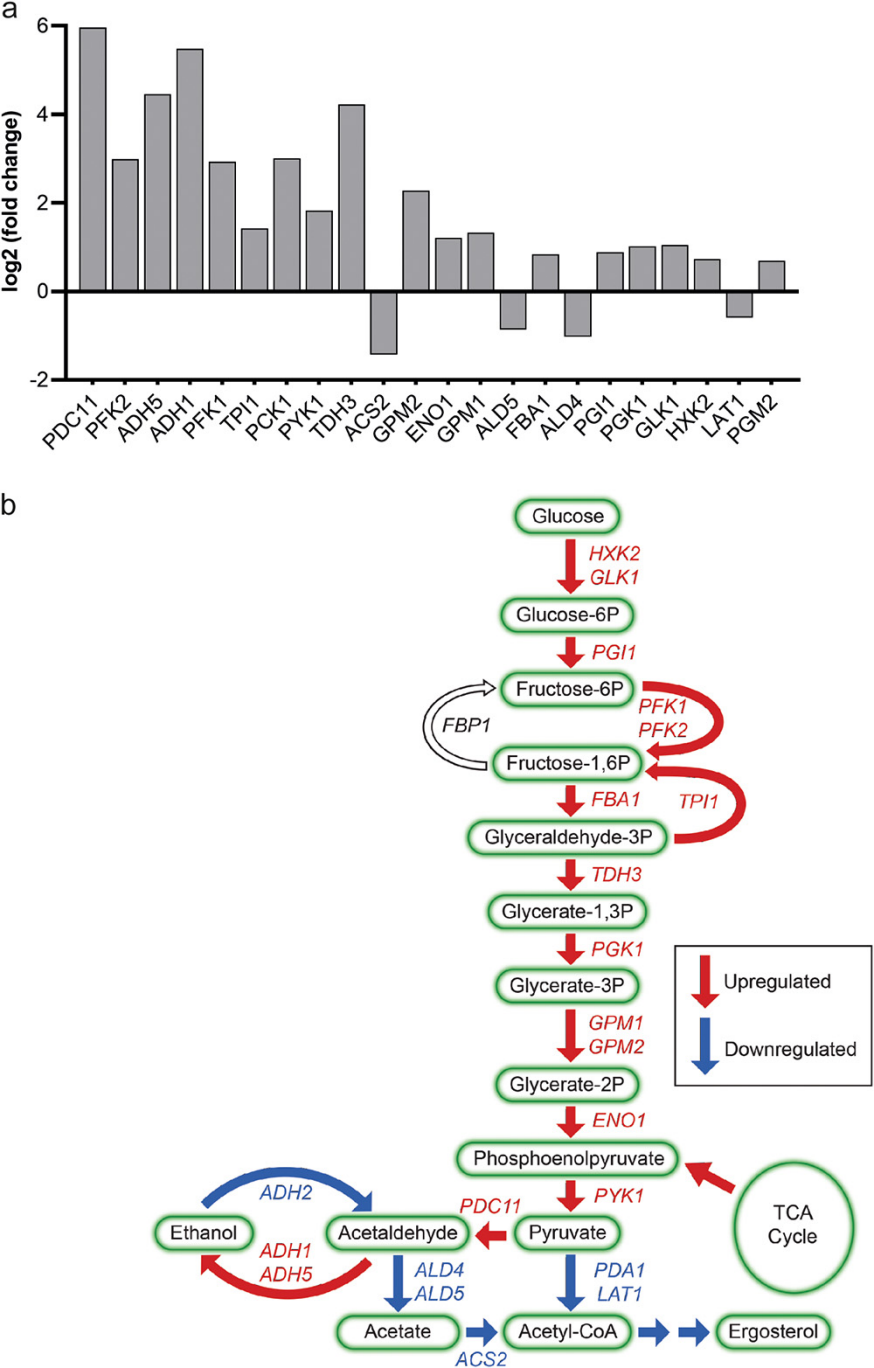
The effects of pyrvinium pamoate on central carbon metabolism. (a) Differentially expressed genes in the glycolysis/gluconeogenesis KEGG pathway. Significantly changed genes with decreasing *P* values left to right (cutoff ≤ 0.01) are presented. The nomenclature of C. auris genes is based on the closest C. albicans homologue. (b) Schemata of carbon metabolism and its links with ergosterol biosynthesis. The arrow indicates the direction of the enzymatic reaction. Upregulated genes are depicted in red and downregulated genes are depicted in blue.

A high number of genes involved in carbon metabolism were differentially expressed in the presence of PP (Fig. 2b). To gain further insight, we mapped differentially expressed genes onto the KEGG database (https://www.genome.jp/kegg/). Notably, PP triggered the upregulation of all genes encoding enzymes in the glycolytic pathway, which convert glucose to pyruvate (Fig. 3a and b). The alcohol dehydrogenases *ADH1* and *ADH5*, whose gene products catalyze the fermentation of pyruvate to ethanol, were also upregulated, as was the homolog of pyruvate decarboxylase *PDC11* that converts pyruvate to acetaldehyde (Fig. 3a and b). In contrast, the pyruvate dehydrogenase genes *PDA1* and *LAT1* were downregulated (Fig. 3a and b). The downregulation of pyruvate dehydrogenase expression indicates that PP may inhibit the conversion of pyruvate into acetyl coenzyme A (acetyl-CoA) for entry into the TCA cycle. The gene of acetyl-CoA synthase Acs2, which catalyzes the formation of acetyl-CoA from acetate, was also downregulated (Fig. 3a and b). In summary, our data suggest that PP could act as an inhibitor of the TCA cycle, with a compensatory upregulation of glycolysis and fermentation of pyruvate to ethanol.

**FIG 4:**
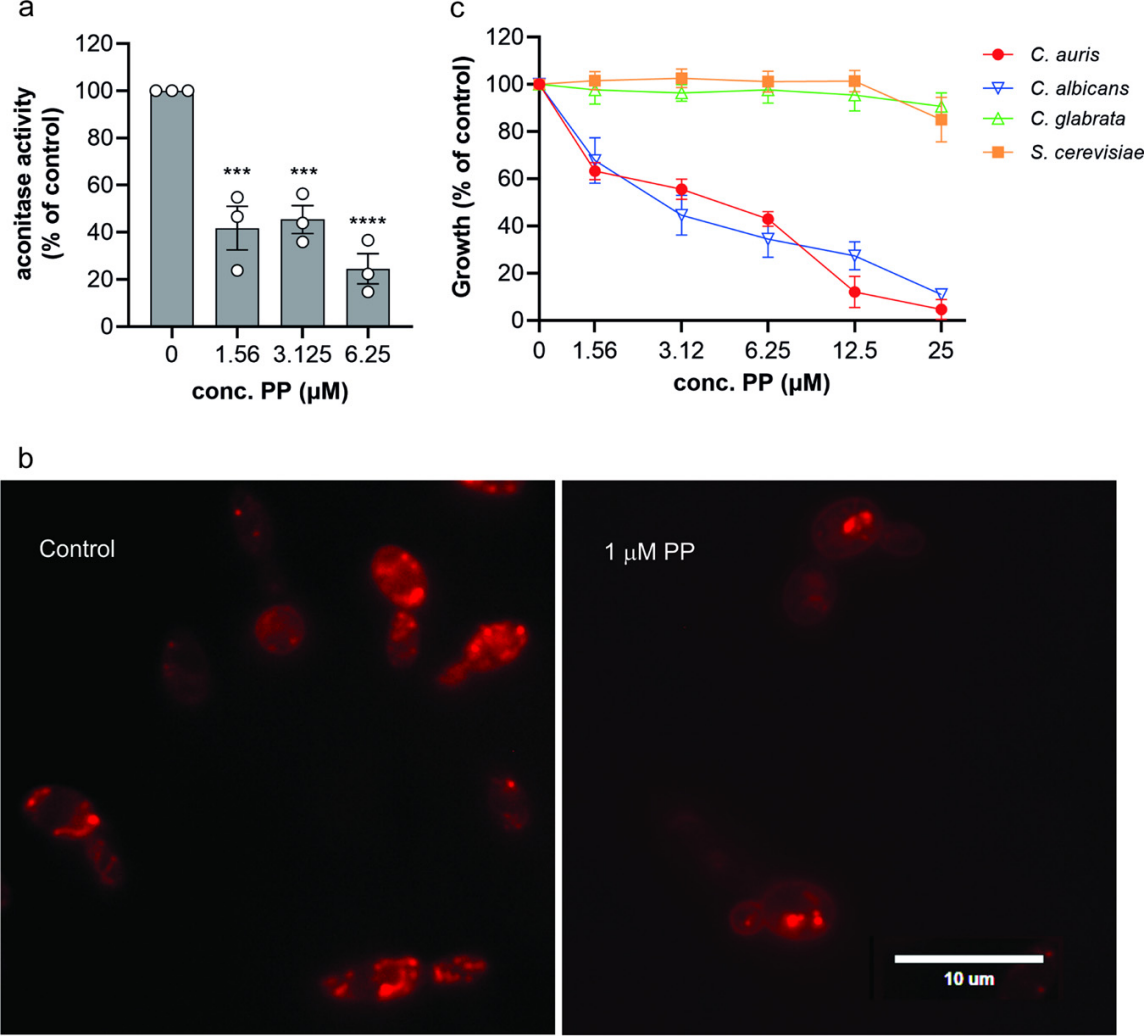
Pyrvinium pamoate inhibits the TCA cycle and induces mitochondrial dysfunction. (a) C. auris cell suspension was adjusted to a cell density of 0.1 and incubated with indicated concentrations of PP. After 20 h of growth at 37°C, cells were harvested and lysed. Aconitase activity was measured and correlated with total protein concentration of the lysate. Error bars indicate SEM with *n* = 3 biological replicates. Each treatment group was compared to control with a one-way ANOVA with Dunnett’s multiple-comparison test (***, *P* ≤ 0.001; ****, *P* < 0.0001). (b) C. auris cells suspension was adjusted to a cell density of 0.1 and incubated without or with 1 μM PP for 6 h at 37°C. Cells were stained with MitoTracker Red CMXRos, and mitochondria were imaged with an Olympus BX60 microscope at ×40 magnification using the DsRed fluorescence channel. The images have been enlarged and cropped. The scale bar has been calibrated accordingly and added manually to the image. The uncropped and unprocessed images are shown in Fig. S7. (c) C. auris (470140), C. glabrata (ATCC 2001), C. albicans (SC5314), and S. cerevisiae (W303) were grown in YNB medium at 30°C according to a modified CLSI-M27 procedure, and cell density at 600 nm was measured after 20 h of growth. The data shown are the means ± SEM (*n* = 4).

To test this hypothesis, we measured the activity of aconitase, the mitochondrial TCA cycle enzyme catalyzing the conversion of citrate to isocitrate. We found a significant downregulation of aconitase activity for all PP concentrations tested (Fig. 4a). Enzyme activity was reduced by 60% compared to control cultures in the presence of 1.56 μM and 3.125 μM PP. A further statistically significant reduction could not be achieved by increasing the PP concentration to 6.25 μM, suggesting a threshold aconitase activity level at which fungal cells are still viable. Indeed, our attempts to obtain sufficient cell material to measure aconitase activity at 12.5 μM PP were unsuccessful, suggesting that cells were inviable at this concentration of PP.

To further address the effects of PP on mitochondria and cellular respiration, we stained C. auris mitochondria with MitoTracker Red. The fluorescence of MitoTracker Red depends on intact mitochondrial membrane potential. In response to PP treatment, MitoTracker staining was reduced (Fig. 4b). In untreated controls, mitochondria stained throughout the cell with characteristic morphology (Fig. 4b). PP treatment caused mitochondrial staining to become localized into a few punctate structures (Fig. 4b). These changes are consistent with PP disrupting both mitochondrial morphology and membrane potential.

**FIG 5:**
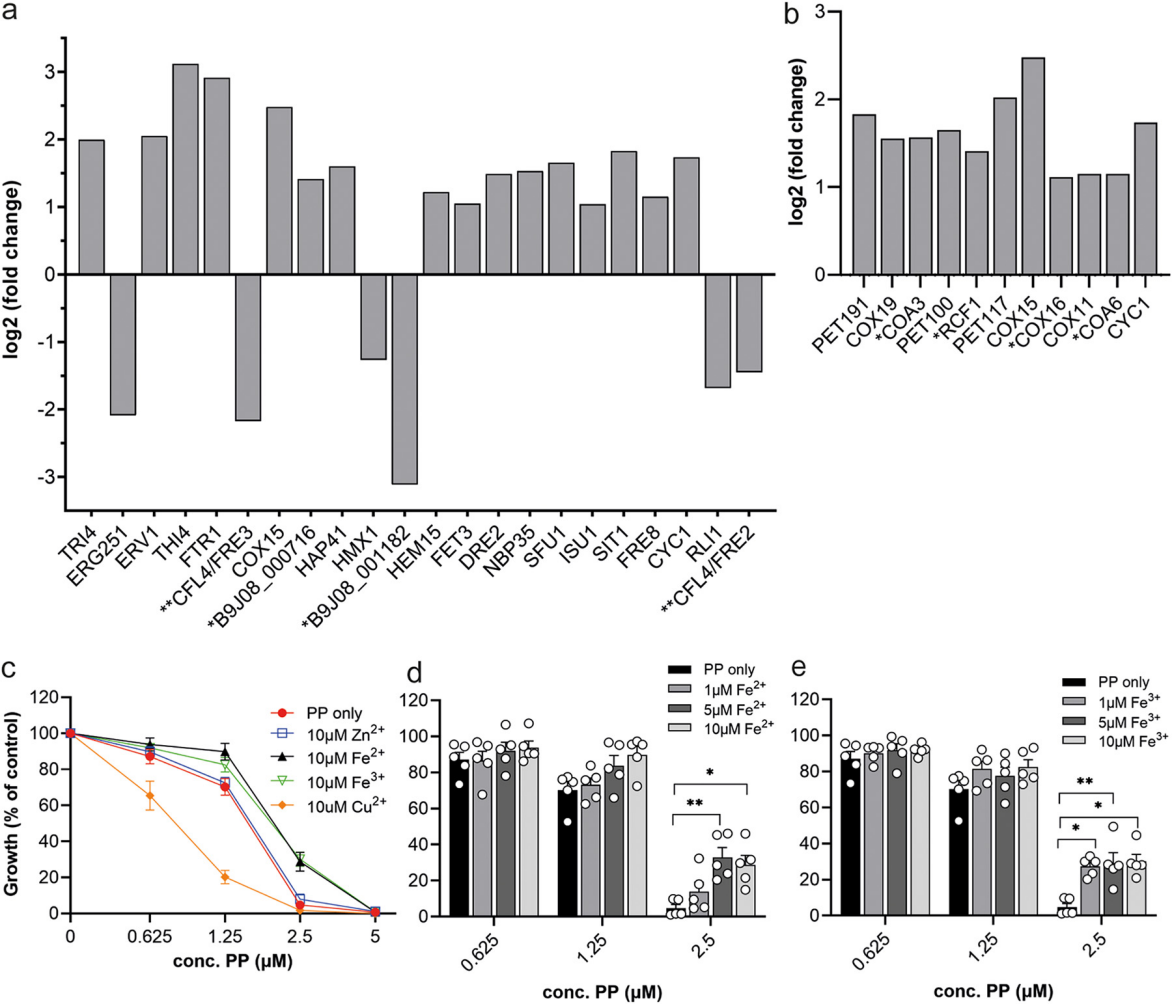
Pyrvinium pamoate impairs iron homeostasis in C. auris. (a) Differentially expressed genes in the iron-binding and transport KEGG pathway. Significantly changed genes with decreasing *P* values left to right (cutoff ≤ 0.05) are presented. Gene nomenclature is based on the closest C. albicans homologue. *, genes have no orthologs in other *Candida* species. **, genes annotated to the same C. albicans but different S. cerevisiae ortholog. (b) Differentially expressed genes in the cytochrome c oxidase catalytic or assembly process. Significantly changed genes with decreasing *P* values left to right (cutoff ≤ 0.05) are presented. Gene nomenclature is based on the closest C. albicans or *S. cerevisiae homologue. (c to e) Iron addition improves growth of C. auris treated with PP. C. auris was grown in RPMI 1640 medium supplemented with metals as indicated: (c) 10 μM zinc, iron, or copper, (d) ferrous iron at the indicated concentrations, or (e) ferric iron at the indicated concentrations. MIC assays were prepared according to CLSI-M27 procedure. Cell density at 600 nm was measured after 20 h of growth at 37°C. The experiments presented in panels c to e with the different metals were performed together but are presented here in separate graphs for clarity. The same data sets for control and 10 μM iron are shown in multiple plots. The data shown are the means ± SEM (*n* = 3 to 5). Each metal treatment group was compared to its corresponding PP only sample with a one-way ANOVA with Tukey’s multiple-comparison test (*, *P* ≤ 0.05; **, *P* ≤ 0.01).

**FIG 6:**
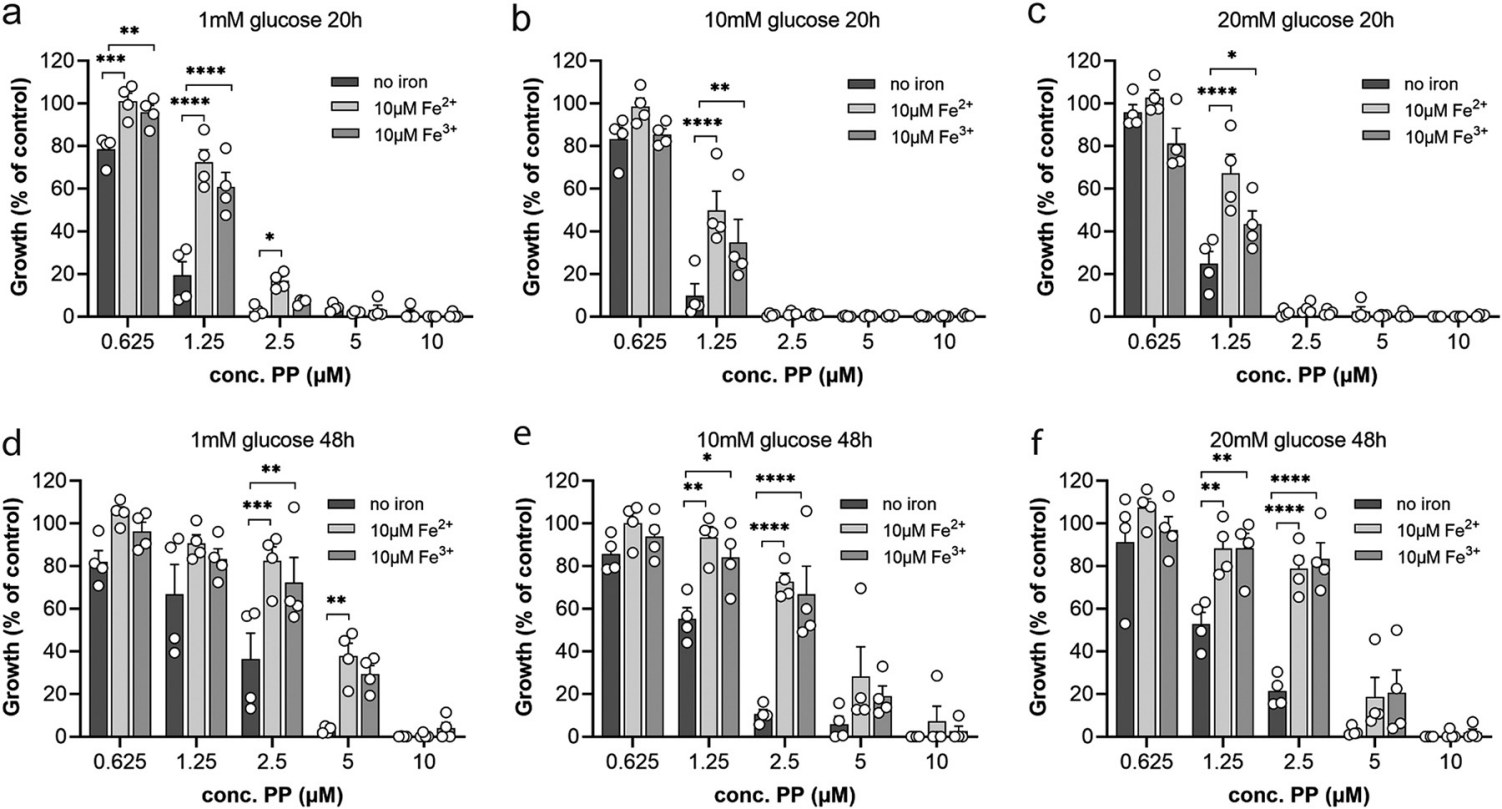
The iron rescue effect is enhanced with time but is little influenced by glucose concentration. C. auris was grown in RPMI 1640 with final concentrations of (a and d) 1 mM, (b and e) 10 mM, or (c and f) 20 mM glucose in absence or presence of 10 μM ferrous or ferric iron. All assays were prepared according to CLSI-M27 procedure. Cell density at 600 nm was measured after (a to c) 20 h and (d to f) 48 h of growth at 37°C. Error bars indicate SEM with *n* = 4 biological replicates. Each treatment group was compared to no-iron controls with a one-way ANOVA with Šídák’s multiple-comparison test (*, *P* ≤ 0.05; **, *P* ≤ 0.01; ***, *P* ≤ 0.001; ****, *P* < 0.0001).

To explore the hypothesis that PP inhibits C. auris by targeting mitochondria, we tested other fungal species with different abilities to tolerate loss of mitochondrial respiration. C. albicans is a so-called “petite-negative” yeast, i.e., it does not tolerate loss of mitochondrial DNA and respiration. Candida glabrata and Saccharomyces cerevisiae are “petite-positive,” meaning they can grow in the absence of mitochondrial DNA and respiration (44, 45). Due to poor growth of S. cerevisiae in RPMI and at 37°C, this experiment was carried out in yeast nitrogen base (YNB) and at 30°C. The petite-positive yeasts C. glabrata and S. cerevisiae were resistant to PP compared to the petite-negative C. albicans (Fig. 4c). No MIC could be observed for C. glabrata and S. cerevisiae for all concentrations of PP tested. Please note that a general shift in susceptibility has occurred due to the change of the assay medium and temperature. The iron content of different media had been measured previously and showed 0.09 μM, 1.82 μM, and 9.25 μM for RPMI, YNB, and YPD, respectively (Claudia Simm, unpublished data), which is in accordance with published results (46). Given that iron can partially rescue fungal growth in PP-treated cultures (Fig. 5 and 6), it can be speculated that the 20-fold excess of iron in YNB compared to that in RPMI medium may account for the difference in the MIC. Our data show that PP is strongly inhibitory for those yeast species that cannot tolerate loss of mitochondrial respiration, while petite-positive yeast grow in the presence of PP. The petite nature of C. auris has not yet been investigated, but its susceptibility to PP and the close phylogenetic distance to C. albicans suggest that it, too, is a petite-negative yeast.

We further explored whether the availability of glucose affects fungal susceptibility to PP, as in mammalian cell lines, the efficacy of PP is exacerbated under glucose starvation (47). However, this was not the case for C. auris (Fig. S3). Neither the concentration of glucose supplemented in the medium nor a switch to mannose altered the susceptibility of C. auris toward PP (Fig. S3a). While growth of C. auris without glucose in the medium was strongly reduced (Fig. S3b), PP did not further exert its activity compared to glucose-supplemented media.

Next, we wanted to test if mitochondrial dysfunction caused by PP could be rescued by ATP supplementation in the medium. ATP did not rescue the growth of PP-treated C. auris at any of the concentrations that we tested, at either 20 h or at 48 h, when a lack of energy supply might be more prevalent (Fig. S4).

### Pyrvinium pamoate interferes with iron homeostasis and iron-dependent metabolism in C. auris.

Our RNA-seq analysis showed that, in addition to metabolic reprogramming, a major effect of PP was on the expression of genes related to metal ion binding and transport (Fig. 2b). Mitochondrial metabolism, respiration, and ergosterol biosynthesis, which were affected by PP as shown above, all depend on iron availability for the synthesis of heme and/or iron-sulfur clusters. For example, the PP-inhibited TCA cycle enzyme aconitase (Fig. 4a) contains an iron-sulfur cluster.

To further understand the effect of PP on iron metabolism in C. auris, we performed a detailed KEGG analysis of iron-binding and transport genes identified in our RNA-seq data. Many genes in these categories were upregulated by PP, with the highest increase seen for genes encoding the iron-binding thiamine enzyme Thi4 and the high-affinity iron permease Ftr1 (Fig. 5a). Iron import by Ftr1 is dependent on the ferric reductase Fre8 and the multicopper ferroxidase Fet3. Both *FTR1* and *FET3* were upregulated by PP. The siderophore-mediated iron uptake transporter *SIT1* was also induced under PP treatment.

Notably, three genes (*CYC1*, *COX15*, and *TRI4*) encoding iron- and/or heme-binding proteins within the mitochondrial electron transport chain were upregulated by PP. *TRI4* encodes a cytochrome P450 protein with a heme-iron center facilitating electron transport. *COX15* encodes the heme a synthase for cytochrome c oxidase, while *CYC1* encodes cytochrome c_1_, which transfers electrons to cytochrome c oxidase. Consistently, several genes encoding cytochrome c oxidase (complex IV) subunits and assembly factors were upregulated by PP treatment (Fig. 5b), suggesting a compensatory mechanism responding to reduced complex IV activity. Moreover, the S. cerevisiae ortholog of essential for respiration and viability (*ERV1*) gene, which has functions in mitochondrial import and iron homeostasis, was one of the induced genes. Increased induction was seen for the gene product of the ORF B9J08_000716. While not characterized in any closely related fungal species, it is assumed to have iron-binding capacity and a function in electron transport. A further four genes (*ISU1*, *NBP35*, *HEM15*, and *DRE2*) encoding proteins with a role in either iron-sulfur cluster assembly or heme biosynthesis showed significant transcriptional upregulation upon PP treatment (Fig. 5a).

*CFL4*, the ortholog of the S. cerevisiae ferric reductases *FRE2* and *FRE3* involved in the reductive iron uptake system, was among the downregulated genes. The biggest downregulation was seen for B9J08_001182 with a predicted function in heme/iron or oxygen binding. The previously discussed pathways affected by PP, ergosterol biosynthesis, and translation also contain iron-binding proteins. PP caused the downregulation of *ERG251* (an iron-binding oxidoreductase involved in ergosterol biosynthesis) and *RLI1* (an iron-sulfur-containing regulator of translation). Lastly, *HMX1*, which is associated with the utilization of hemin-iron, showed a small but significant decrease in expression (Fig. 5a).

Based on these results, we hypothesized that an important mechanism of action for PP in C. auris is disruption of iron homeostasis, which then alters carbon and lipid metabolism and mitochondria due to the requirement for iron-containing cofactors in enzymes of these pathways. Disruption of iron homeostasis by PP explains the compensatory upregulation of iron assimilation pathways that we observed.

To test this hypothesis, we asked if the addition of metals to C. auris cultures could attenuate the effect of PP. We added 10 μM ferrous or ferric iron to the culture medium with increasing concentrations of PP as well as copper or zinc to address specificity. As seen in Fig. 5c, only the addition of Fe^2+^ and Fe^3+^ could elevate growth of C. auris under PP treatment compared to that in conditions where no metal was added. No change in growth was found for zinc, whereas the addition of copper caused a further decrease of growth compared to the no-metal control. To further investigate the growth rescue by iron, we performed dose titration experiments with Fe^2+^ (Fig. 5d) and Fe^3+^ (Fig. 5e). While improved growth could be observed by adding 5 μM and 10 μM concentrations of either ferrous or ferric iron, at the lower dose of 1 μM, only the addition of Fe^3+^ was enough to show a significant increase in survival of C. auris (Fig. 5e).

In this study, we showed that PP caused mitochondrial dysfunction with an upregulation in glycolysis (Fig. 3) and reduced enzyme activity in the TCA cycle (Fig. 4a and b). We therefore tested if the iron rescue effect would change in response to different glucose concentrations in the medium. We observed an iron rescue effect for all glucose concentrations at both 20 and 48 h (Fig. 6). The supplementation of iron into low-glucose medium resulted in increased growth for PP concentrations lower than 5 μM at 20 h (Fig. 6a) compared to higher glucose concentrations at this time point (Fig. 6b and c). The enhanced protective effect of supplemental iron in low glucose was also evident at 48 h, where growth in 1 mM glucose was significantly increased up to 5 μM PP (Fig. 6d). In general, iron supplementation was also more beneficial at 48 h than at 20 h for 10 mM and 20 mM glucose (Fig. 6b, c, e, and f). Significantly better growth upon iron addition was seen at 1.25 μM PP at 20 h, which extended to 1.25 μM and 2.5 μM PP at 48 h. A trend toward higher cell density was seen at 5 μM PP with iron supplementation at 48 h; hardly any growth was observed at 20 h even with iron addition, suggesting a more urgent need for iron supplementation after an extended growth period.

### Pyrvinium pamoate reduces proliferation of C. auris in a host niche.

PP is an FDA-approved anthelmintic drug and is safe to use in human even at high dosage of 350 mg per tablet (37). We therefore tested if PP could inhibit proliferation of C. auris using two *in vivo* models, Galleria mellonella larvae and murine bone marrow-derived macrophages (BMDMs). PP was well-tolerated by *Galleria*, but it did not rescue from C. auris infection (Fig. S5). The lack of PP efficacy could be due to either poor absorption or the requirement for iron for the antimicrobial activity of the hemolymph of *Galleria*, as demonstrated by the effect of a metal chelator in delaying the removal of the bacteria Xenorhabdus nematophila and Bacillus subtilis from the infected host (48). PP as a cyanine dye has metal chelating capability (49), which may explain why *Galleria* might not be a suitable model for studying compounds that affect iron. Therefore, we next turned to a mammalian model.

**FIG 7:**
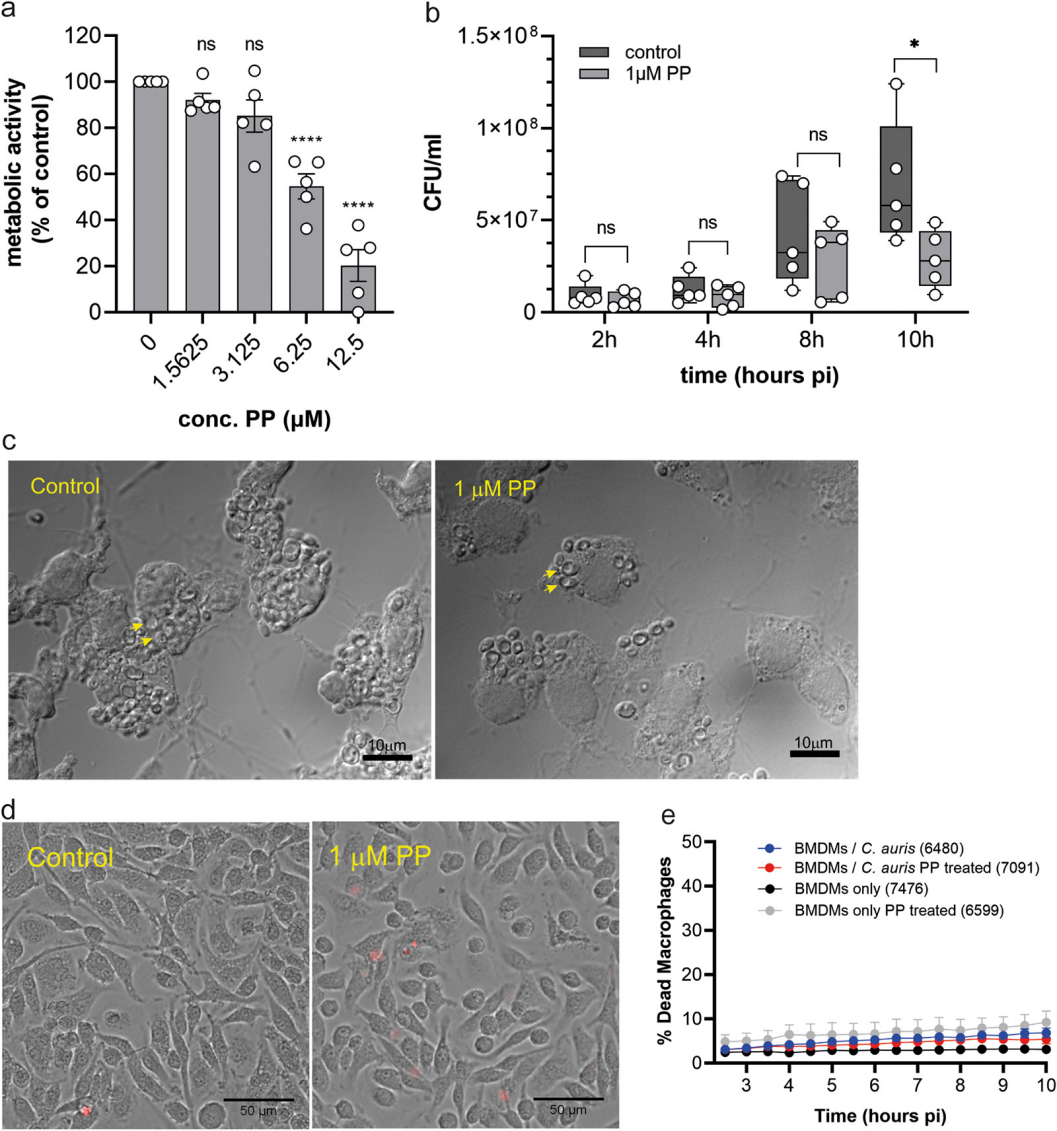
Pyrvinium pamoate inhibits growth of C. auris in a host niche. (a) Uninfected bone marrow-derived macrophages (BMDMs) were treated with various concentrations of PP for 20 h. Viability was measured using the resazurin metabolic assay. Error bars indicate SEM with *n* = 5 biological replicates. Each treatment group was compared to the control with a one-way ANOVA with Dunnett’s multiple-comparison test (ns, *P* ≥ 0.05; ****, *P* < 0.0001). (b) BMDMs were infected with C. auris at an MOI of 2:1 in presence or absence of 1 μM PP. Supernatant was removed and macrophages were lysed at 2 h, 4 h, 8 h, and 10 h postinfection (p.i.). Released C. auris were plated on YPD plates. CFU were counted after 2 days of incubation at 30°C. The box plot shows 5 independent experiments and their median values. Ratio-paired two-tailed *t* test was performed, with ns, *P* ≥ 0.05 and *, *P* = 0.015. (c) Microscopy images of BMDMs infected with C. auris without and with 1 μM PP treatment at 10 h p.i. at a ×100 magnification. Yellow arrows indicate phagocytosed C. auris. (d and e) Live-cell imaging of infected (MOI 3:1) and uninfected BMDMs without and with 1 μM PP treatment. (d) Magnified view of DRAQ7 staining of uninfected BMDMs with or without PP treatment at 10 h p.i. For uncropped image, see Fig S6. (e) Cumulative macrophage death (DRAQ7-positive BMDMs) was calculated from live-cell imaging. Graph shows mean values and SEM of three biological with two technical repeats. The number in brackets indicates the number of macrophages assessed per treatment group.

Macrophages play important roles in host defenses against fungi and have recently been implicated in defenses against C. auris (50, 51). C. auris rapidly proliferates inside macrophages, showing that this is a relevant replicative niche in the host (51). First, we determined if PP was tolerated by macrophages. After 20 h of treatment, PP concentrations of 1.5 μM and 3.1 μM did not affect macrophages, while at 6.25 μM and 12.5 μM PP, macrophages showed decreased metabolic activity (Fig. 7a). For all subsequent experiments with BMDMs, a concentration of 1 μM PP was used.

BMDMs were challenged with C. auris at a multiplicity of infection (MOI) of 2:1 (yeast/macrophages) for 1 h. After removing nonphagocytosed C. auris, macrophages were lysed for the release of internalized yeast at 2, 4, 8, and 10 h postinfection (p.i.) and CFU were counted. Consistent with the study by Bruno et al., C. auris replicated inside macrophages (Fig. 7b) (51). A trend toward lower C. auris cell numbers inside BMDMs for the PP-treated samples was visible at 8 hours p.i. with a significant difference at 10 hours p.i. (Fig. 7b). At this time point, the median CFU count in the treated culture was 2-fold lower than that of the control. This is in accordance with our *in vitro* results, in which 1 μM marks the IC_50_ value for PP against C. auris.

Microscopy also confirmed the decrease in internalized C. auris in the presence of PP compared to that in the control condition (Fig. 7c). High numbers of C. auris were visible inside BMDMs (yellow arrows) without PP treatment, and yeast numbers were greatly decreased when 1 μM PP was added to the culture (Fig. 7c). While our metabolic data in Fig. 7a show that uninfected macrophages are not susceptible to PP at antifungal concentrations, we also wanted to test if this was the case for infected macrophages. Therefore, we used imaging to address the viability of infected macrophages by staining dead cells with the dye Draq7. Imaging of uninfected macrophages was consistent with the metabolic data shown in Fig. 7a, in that macrophages maintained their cell integrity in the presence of PP during the 10 h of the assay (Fig. 7d and e and Fig. S6). C. auris-infected macrophages also maintained cell viability in the presence of PP (Fig. 7e). In fact, little cell death was seen for either C. auris-infected or uninfected macrophages with or without PP treatment (Fig. 7e) in the time frame of the assay. Collectively, our data suggest that PP reduces C. auris proliferation inside macrophages in the first 10 h postinfection in a fungal-selective manner.

## DISCUSSION

Our study establishes metabolism as a viable target against the drug-resistant fungal pathogen C. auris, demonstrating that the growth of this drug-resistant pathogen is inhibited by pyrvinium pamoate (PP) by a multipronged metabolic mechanism that disrupts iron, carbon, and lipid metabolism and thereby compromises the utilization of both macro- and micronutrients. We provide detailed insight into the antifungal mechanism of action of PP, showing that it causes wide-spread metabolic shifts and mitochondrial dysfunction. Our data show that petite-positive yeast species S. cerevisiae and C. glabrata are much less susceptible to PP than petite-negative C. albicans and C. auris, supporting a role for mitochondria in PP’s mechanism of action. Furthermore, our data support the hypothesis that the metabolic dysfunction caused by PP is strongly interconnected to its effect in disrupting iron homeostasis. Notably, the antifungal activity of PP is conserved across C. auris clades, although unlike in other fungal species, PP does not synergize with fluconazole. PP inhibited the replication of C. auris in macrophages without affecting immune cell metabolism at antifungal concentrations, showing it can act in a fungal-selective manner.

Our extensive investigations into the transcriptional response of C. auris to PP identified significant changes in ion homeostasis and carbohydrate metabolism, specifically glycolysis, pyruvate metabolism, and ATP generation. Genes associated with glycolysis and fermentation were upregulated by PP, while those associated with the generation of acetyl-CoA from pyruvate or acetate were downregulated. Acetyl-CoA is critical for driving the TCA cycle in the mitochondria, and we show that the activity of aconitase, a key TCA cycle enzyme, was lower in the presence of PP. We propose that the upregulation of glycolysis by PP serves to compensate for compromised mitochondrial metabolism. Imaging of C. auris mitochondria upon staining with MitoTracker Red CMXRos showed that PP caused reduced staining and a staining pattern more fragmented than that of control conditions (Fig. 4B). This result is consistent with PP causing mitochondrial dysfunction due to loss of mitochondrial membrane potential and fragmentation of mitochondria (52, 53). Indeed, many genes encoding cytochrome oxidase subunits or factors involved in the assembly of this heme complex were upregulated in PP-treated C. auris (Fig. 5b), presumably to compensate for defects in the electron transport chain. We also show that petite-positive yeasts C. glabrata and S. cerevisiae are resistant to PP at all concentrations tested, whereas C. auris and the petite-negative yeast C. albicans are susceptible (Fig. 4c). This indicates that PP becomes ineffective in yeast species that do not rely on mitochondrial respiration in the presence of fermentable carbon sources such as glucose. Similar to our conclusions, a compound targeting the C. albicans mitochondrial phosphate carrier was effective against C. auris but ineffective against the petite-positive S. cerevisiae and C. glabrata in the presence of glucose (54), which is consistent with the need for respiratory metabolism in the antifungal mechanism of action of at least some mitochondria-targeting compounds. Taken together, our data are consistent with PP exerting its antifungal activity by compromising mitochondrial respiration and metabolism. To our knowledge, this is the first time that mitochondrial dysfunction has been described as the mechanism of action of PP in any fungal pathogens.

Mitochondria are key organelles for fungal iron metabolism. Many proteins involved in mitochondrial processes such as electron transport or the TCA cycle have either an iron-heme or an iron-sulfur cluster cofactor (55). Iron import is facilitated mainly through Fet3/Ftr1 or Sit1 transporters. Remarkably, C. auris contains an expansion of *SIT1*-like genes of up to 14 orthologues compared to 1 in C. albicans, indicating its greater need for this metal but also revealing an Achilles heel (36). Our data indicate that the way PP compromises mitochondria in C. auris is interwoven with disrupting iron metabolism. Supporting this conclusion, PP reduces intracellular iron levels in the fungal pathogen C. albicans, where zinc levels were also affected, albeit at higher PP concentrations (56). By showing that supplementation with iron, but not zinc or copper, rescues C. auris from PP (Fig. 5c to e), we demonstrate that iron metabolism is altered in response to PP in C. auris. Consistently, genes encoding iron uptake, such as *FET3*, *FTR1*, and *SIT1*, are significantly upregulated by PP, and proteins that require iron as cofactors, such as aconitase in the TCA cycle, show reduced enzymatic activity (Fig. 5a and Fig. 4a). Unlike iron, the supplementation of ATP did not improve the growth of PP-treated C. auris (Fig. S4). While there is no experimental evidence that C. auris is able to import ATP from the extracellular environment and only an uncharacterized ORF (B9J08_000434) that may encode an ATP:ADP antiporter could be identified in its genome, other yeasts, such as C. albicans and S. pombe, are able to utilize external ATP (57, 58). Combinational assessment of elevated iron in different glucose concentration revealed that glucose concentration had little effect on the iron rescue effect (Fig. 6). In other words, upon PP treatment, fungal growth could be rescued significantly by iron addition but not by an increase in glucose levels or ATP. While mitochondria activity and iron are closely connected functionally and it is therefore difficult to know precisely where the primary activity of PP lies, our data with supplementation of metabolites indicated that iron homeostasis might be the primary target of PP, with metabolic and mitochondria disruption following from that.

While we did not observe significant transcriptional changes of enzymes in the TCA cycle, reduced enzymatic activity of the Fe-S cluster enzyme aconitase coupled with a downregulation of genes encoding acetyl-CoA-synthesizing enzymes suggests a lower metabolic rate for this cellular process (Fig. 3 and Fig. 4a). Acetyl-CoA is a precursor for ergosterol synthesis, a metabolic pathway that also critically depends on iron. For example, the azole drug target Erg11 contains heme as a cofactor. Indeed, iron deficiency and mitochondrial dysfunction have been linked to increased sensitivity toward azoles (59). Our data show that many genes in the ergosterol pathway were significantly downregulated in PP-treated C. auris, and checkerboard experiments revealed that the azole fluconazole, and to a lesser extent the echinocandin caspofungin, potentiated the effect of PP in an additive manner (Fig. S2). The interaction of antifungals with PP in C. auris is weaker than that described in C. albicans, Aspergillus fumigatus and Exophiala dermatitidis were azoles, and PP acted synergistically (60–62). We speculate that the reduced nature of potentiation in C. auris may be a result of its intrinsic resistance to azole antifungals.

PP has been studied as an anticancer drug with a range of proposed mechanisms, including inhibition of complex I or II in the mitochondrial electron transport chain (63, 64), interference with glucose metabolism, STAT3 signaling, and the inhibition of NADH-fumarate reductase (63, 65–67). We show that PP triggers the disruption of iron homeostasis in C. auris and causes major metabolic reprogramming, reduced activity of TCA cycle enzymes, and structural changes in mitochondria which could result from a failure to assemble heme and Fe-S cluster proteins. As such, our results might be informative for the anticancer mechanism of PP, as it is tempting to speculate that disruption of iron homeostasis by PP is one of the driving modes of action. However, differences exist between fungal and mammalian systems. The cytotoxicity of PP against cancer cells and cardiac fibroblast was dramatically increased in low-glucose conditions (47, 63, 68), consistent with higher reliance on glycolysis when mitochondria are inhibited (the so-called Warburg effect) (69). Genes involved in glycolysis and pyruvate fermentation were upregulated by PP in C. auris (Fig. 3), which is reminiscent of the Warburg effect in mammalian cells (70). However, in contrast to mammalian cells, changes in carbon source or glucose concentrations had no effect on the efficacy of PP against C. auris (Fig. S3). Taken together, these data indicate differences in metabolic flexibility between C. auris and mammalian cells when it comes to overcoming the metabolic dysfunction caused by PP. This conclusion is also supported by our macrophage infection experiments (Fig. 7). The rapid growth of C. auris inside BMDMs was significantly reduced by PP at 10 h postinfection at concentrations that did not compromise the metabolic activity or viability of macrophages in the same time frame.

In conclusion, our study has shown that PP perturbs fungal metabolism at the micronutrient and the macronutrient level by disrupting iron homeostasis and carbon and lipid metabolism and causing mitochondrial changes. These metabolic perturbations ultimately reduce growth of the multidrug-resistant pathogen C. auris. This “two-hit” metabolic disruption might be especially effective in host niches by potentiating nutritional immunity through iron deprivation and further restricting fungal metabolic flexibility that is needed in glucose-poor host niches, such as the macrophage phagosome. The fact that macrophages are more resistant to PP than C. auris shows that metabolic differences between host and pathogen might be exploitable for designing well-tolerated antifungal therapies. Anticancer therapies exploit the distinct metabolism of cancer cells, and we now show that a similar approach could be used for eukaryotic pathogens which, like cancer cells, share drug targets with their host. As such, our study sheds light on targeting metabolism and mitochondria as a promising therapeutic strategy against the worrisome drug-resistant pathogen C. auris.

## MATERIALS AND METHODS

### *Candida* strains and media.

*Candida* and S. cerevisiae isolates and strains used in this study are listed in Table S1. The C. auris isolates 470121 and 470140 were a generous gift from Sarah Kidd (National Mycology Reference Center, Adelaide), and the other isolates were obtained from the CDC (Atlanta, USA). All isolates were maintained on YPD plates (1% yeast extract, 2% peptone, 2% glucose, and 2% agar). When precultures were required, single colonies of C. auris were picked from YPD plates, inoculated into liquid YPD medium, and grown at 30°C for 20 h. Unless stated otherwise, all experiments were carried out in serum-free RPMI 1640 medium (R6504, Sigma), buffered with 3.5% MOPS (morpholinepropanesulfonic acid), and adjusted to pH 7. For experiments investigating different C-sources, RPMI 1640 medium (R1383, Sigma) with 3.5% MOPS was used supplemented with glucose (0, 10, 20 mM) or mannose (10 mM). Growth experiments with petite-positive and petite-negative yeasts were carried out in YNB supplemented with 0.2% yeast synthetic dropout mix.

### MIC and synergism experiments.

Experiments were set up according to the broth microdilution adaptation of CLSI-M27. Pyrvinium pamoate (PP) powder (P0027, Sigma) was dissolved in 100% dimethyl sulfoxide (DMSO) to achieve a stock concentration of 10 mM. PP stock solution was diluted in the appropriate medium to 2-fold concentrated (MIC) or 4-fold concentrated (synergism experiment). *Candida* and S. cerevisiae inocula were prepared as described in CLSI-M27. In brief, 5 yeast colonies were suspended in phosphate-buffered saline (PBS) and adjusted to an optical density at 600 nm (OD_600_) of 0.08 to 0.1. A volume of 100 μL of cell suspension was added to 9.9 mL RPMI 1640 or YNB medium to make the working solution. Then, 50 μL of PP and 50 μL of working solution were added into flat-bottom 96-well plates and incubated either at 37°C for 20 h or for growth assays in YNB at 30°C for 20 and 48 h. For synergism experiments, 25 μL of PP dilutions, 25 μL of antifungal drugs (caspofungin, fluconazole, and amphotericin B), and 50 μL of working solution were mixed and incubated at 37°C for 20 h. Cell density at 600 nm was measured using a plate reader (Tecan, Spark 10 M). The IC_50_ values in Table 1 were calculated using a four-parameter nonlinear regression equation (Prism 9, GraphPad) utilizing the Hill equation,

$$\text{y}=\min+\frac{\max-\min}{1+\left(\frac{x}{IC_{50}}\right)-\mathrm{hillslope}}$$

Synergistic effects were evaluated using the fractional inhibitory concentration index (FICI) method, whereby MIC_A_ and MIC_B_ are defined as the MICs of each drug alone and MIC_AC_ and MIC_BC_ are the corresponding MICs of both drugs in combination, $\mathrm{FICI}=\frac{{\mathrm{MIC}}_{\mathrm{AC}}}{{\mathrm{MIC}}_{A}}+\frac{{\mathrm{MIC}}_{\mathrm{BC}}}{{\mathrm{MIC}}_{B}}$. Drug interactions with FICI values of <0.5, >0.5 to <1, 1 to 4, or >4 are categorized as synergistic, additive, indifferent, and antagonistic, respectively.

### CFU/experiments.

C. auris precultures were grown in RPMI 1640 medium for 18 to 20 h overnight. The next morning, cultures were adjusted to a cell density of 0.1 (1 × 10^6^ cells) and treated with various concentrations of PP as indicated in the figure legends. Cells were exposed to the drug for 15, 30, 45, or 60 min for optimization of RNA-seq conditions or for 20 and 48 h to determine fungicidal versus fungistatic properties before being diluted and plated onto YPD plates. CFU were counted after a 2-day incubation at 30°C.

### Macrophage infections and live-cell imaging experiments.

Bone marrow-derived macrophages (BMDMs) from mice were prepared as previously described (33), by extracting bone marrow from the femur and tibia bones of 6- to 8-week-old mice (strain C57BL/6) and then differentiating into macrophages in BMDM medium that contains RPMI 1640 medium, 12.5 mM HEPES, 20% l-cell conditioned medium, 15% fetal bovine serum, and 100 U/mL of the antibiotics penicillin-streptomycin. Macrophages were left to differentiate for 6 to 8 days at 37°C and 5% CO_2_. All experiments using mice for the isolation of macrophages were approved by the Monash University Animal Ethics Committee (approval number MARP-2015-170/ID 14292 and ERM25488).

For infection experiments, BMDMs were counted in a hemacytometer and seeded at 2.5 × 10^5^ cells per well in a 24-well plate and incubated at 37°C and 5% CO_2_ overnight. The next day, macrophages were coincubated with the C. auris isolate 470140 (multiplicity of infection [MOI] 2:1 *Candida*/macrophages) without drug addition. After 1 h of coincubation, nonphagocytosed C. auris cells were removed by washing three times in PBS, and then prewarmed BMDM medium with or without 1 μM PP was added to the infected macrophages, which were incubated for a further 9 h. Images were taken with an Olympus BX60 microscope at ×100 magnification and further cropped and adjusted for brightness.

For live-cell imaging, BMDMs were seeded at 5 × 10^4^ cells per well in a 96-well plate in BMDM medium and incubated overnight at 37°C and 5% CO_2_. BMDMs were stained with 1 μM CellTrackerGreen CFMDA dye (ThermoFischer C7025) in serum-free BMDM medium as described previously (71). Macrophages were then infected with C. auris at an MOI of 3:1 C. auris/macrophages in BMDM medium for 1 h, after which all nonphagocytosed yeasts were removed by washing three times in PBS. Fresh medium with or without 1 μM PP was added and all cells were stained with 0.6 μM DRAQ7 (Abcam). The 96-well plate was put into an incubation chamber and time-lapse images were acquired with a Leica AF6000 LX epifluorescence microscope every 30 min for up to 24 h using a 10×/0.8-A objective with bright field, green fluorescent protein (GFP), and Y5 filters. For data analysis, the MetaMorph (Molecular Devices) was used as described in Tucey et al. (33). DRAQ7-positive events were plotted using Prism 9 (GraphPad) software.

For the quantification of fungal CFU, at the time points indicated in the figure legend, BMDM medium was removed and macrophages were lysed with ice-cold distilled water to release internalized C. auris. The cell suspension was diluted and plated onto YPD plates. After 2 days of incubation at 30°C, CFU were counted.

### RNA-seq experiment.

For RNA-seq experiments, C. auris precultures were grown in RPMI 1640 medium for 18 to 20 h. Precultures were diluted to a cell density of 0.1 in fresh RPMI, and cultures were grown in the absence or presence of 1 μM PP for 30 min. Cells were harvested and snap-frozen. RNA was extracted using the hot phenol method, and the poly(A)-test RNA-sequencing (PAT-seq) library was prepared as described previously (72). The experiment was performed 3 independent times.

The raw sequencing data from three biological independent experiments were processed by the Tail Tool pipeline (https://github.com/Monash-RNA-Systems-Biology-Laboratory/tail-tools) as described previously (73). The poly(A) sequence was removed from reads, which were then mapped onto the C. auris B8441 sequence assembly: http://www.candidagenome.org/cache/C_auris_B8441_genomeSnapshot.html. Read counts were produced based on reads aligning to annotated genes. To allow for unannotated 3′ untranscribed regions (UTRs), reads up to 400 bases downstrand of a gene but not extending into another gene on the same strand were also counted as belonging to a gene. Differentially expressed genes (DEGs) were selected using the Topconfects package (74) and filtered with a false-discovery rate (FDR) of 0.01 and log_2_ fold change of 1 (Table S2). The FDR represents *P* values adjusted for multiple testing using the Benjamini-Hochberg procedure (75). An interactive data analysis interface is provided at https://degust.erc.monash.edu/degust/compare.html?code=3ca3c1202b154234cab0544358026de0#/.

Gene Ontology (GO) enrichment using the PANTHER classification tool (http://www.pantherdb.org) was performed to identify enriched biological processes and molecular function. DEGs were further searched against the Kyoto Encyclopedia of Genes and Genomes (KEGG) database (https://www.genome.jp/kegg/mapper/) to reveal enriched pathways.

### Resazurin viability assay.

BMDMs were seeded at 5 × 10^4^ cells per well in a 96-well plate in BMDM medium and incubated at 37°C and 5% CO_2_ overnight. The next day, 2-fold dilutions of PP were added to the cells in a volume of 100 μL and incubated at 37°C and 5% CO_2_ for 20 h followed by the addition of 10 μL 10× resazurin. Cells were further incubated at 37°C and 5% CO_2_ until reduction of resazurin was visible (2 to 4 h). Fluorescence (Ex/Em 535/585) was measured using a plate reader (Tecan, Spark 10 M).

### Aconitase assay.

Aconitase activity was measured with an aconitase activity assay kit (MAK051, Sigma). C. auris precultures were grown in RPMI 1640 medium for 18 to 20 h. Precultures were diluted to a cell density of 0.1 in fresh RPMI and treated with various concentrations of PP for 20 h. Cells were harvested and the pellet was resuspended in 100 μL of aconitase assay buffer. After 0.2 g of glass beads was added, cells were lysed in three pulses (40 s) in a bead beater (Savant Fast Prep FP120) with a 30-s recovery on ice between pulses. Glass bead and nonlysed cell material were removed by centrifugation in a tabletop centrifuge (13,000 rpm at 4°C for 5 min). The protein concentration was determined using a bicinchoninic acid (BCA) protein assay kit (Pierce). Fifty microliters of lysate was used for the aconitase activity assay following manufacturer procedures. Enzyme activity was normalized to protein concentration.

### Mitochondrial staining and imaging.

To observe mitochondrial morphology, we inoculated cells from stationary overnight cultures into fresh RPMI medium at a cell density of 0.01 with or without 1 μM PP treatment. After 6 h of treatment, staining was performed with a final concentration of 0.1 μM MitoTracker Red CMXRos in the growth medium. Cells were stained for 30 min at 30°C. Cells were washed three times with 1× PBS prior to microscopy. Images were taken with EVOS FL AUTO imaging system (×40 magnification, DsRed channel). In the figures, representative cells from the micrographs cropped and adjusted for brightness and contrast are shown.

### Galleria mellonella infection.

G. mellonella larvae were grown in-house and kept at 30°C in the dark until the start of the experiment. A colony of C. auris 470140 was streaked out onto YPD plates. The next day, colonies were picked and suspended in PBS. Cell density was adjusted to 5 × 10^6^ cells/mL, and G. mellonella larvae were injected with 5 × 10^4^ cells/larva of C. auris with or without concentrations of PP as indicated in the figure legend. Larvae were incubated at 37°C in the dark, and survival was checked daily for 6 days.

### Data availability.

The RNA-seq data have been deposited in GEO under the submission number GSE176354. The data are also available in interactive form at https://degust.erc.monash.edu/degust/compare.html?code=3ca3c1202b154234cab0544358026de0#/. Data set S1 shows the numerical data used to construct the graphs shown in the figures.
