# Supplementary material for: Disruption of Iron Homeostasis and Mitochondrial Metabolism Are Promising Targets to Inhibit Candida auris
Source: Microbiol Spectr. 2022 Apr 12;10(2):e00100-22. doi: 10.1128/spectrum.00100-22 (PMC9045333; doi:10.1128/spectrum.00100-22)
Supplement: SUPPLEMENTAL FILE 1 — Supplemental material. Download SPECTRUM00100-22-s001.pdf, PDF file, 5.9 MB [file spectrum00100-22-s001.pdf]

Supplementary Figure 1

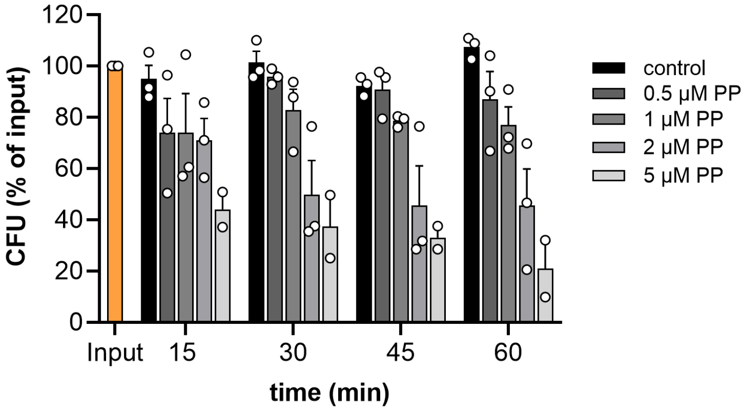

Supplementary Figure 1: **Optimization of pyrvinium pamoate treatment conditions for the RNAseq experiment.** *C. auris* was inoculated at a cell density of 0.1 in RPMI-16040 medium and treated with the indicated concentrations of PP. After 15, 30, 45 and 60 minutes of growth at 37°C aliquots of cultures were diluted and plated onto YPD plates. CFUs were counted after 2 days of incubation at 30°C. The data shown are the means ± SEM (n = 2-3).

Supplementary Figure 2

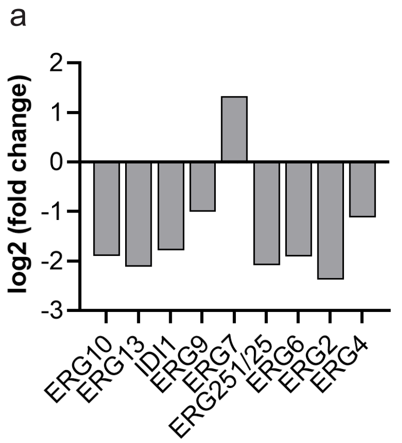

e

FICI	Conc. PP (µM)			
	**0.3125**	**0.625**	**1.25**	**2.5**
**4 µg/ml Flu**	0.66	0.86	0.78	1
**8 µg/ml Flu**	0.7	0.8	0.82	1.2
**1.25 µg/ml Casp**	1.28	1.45	0.71	1.11
**1.5 µg/ml Casp**	1.33	1.4	0.78	1.18
**0.25 µg/ml AmpB**	1	1.15	1.04	0.93
**0.5 µg/ml AmpB**	1.55	1.58	1.42	1.05

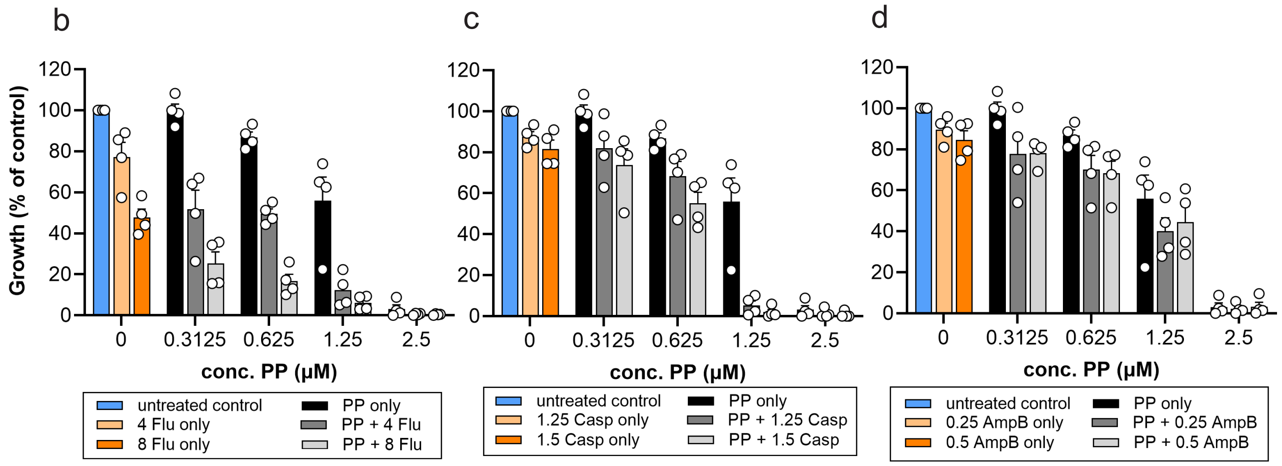

Supplementary Figure 2: **Investigation of synergistic interaction of pyrvinium pamoate with known antifungals. a)** PP downregulates genes in the ergosterol biosynthesis pathway. Genes are sorted according to their position in the ergosterol pathway from left to right. Significantly changed genes with $p < 0.05$ are presented. *C. auris* was inoculated in RPMI-1640 according to the CLSI-M27 protocol and treated with PP at the indicated concentrations and either **b)** fluconazole (Flu) at 4 or 8 µg/ml **c)** caspofungin (Casp) at 1.25 or 1.5 µg/ml or **d)** amphotericin B (AmpB) at 0.25 or 0.5 µg/ml was added in combination with PP. After 20h of incubation at 37°C cell density was measured at 600nm. Error bars indicate SEM with n = 4 biological replicates. **e)** The fractional inhibitory concentration index (FICI) for each drug combination with FICI values indicating > 0.5 – 1 (additive) and > 1 to < 4 (indifferent) interactions.

Supplementary Figure 3

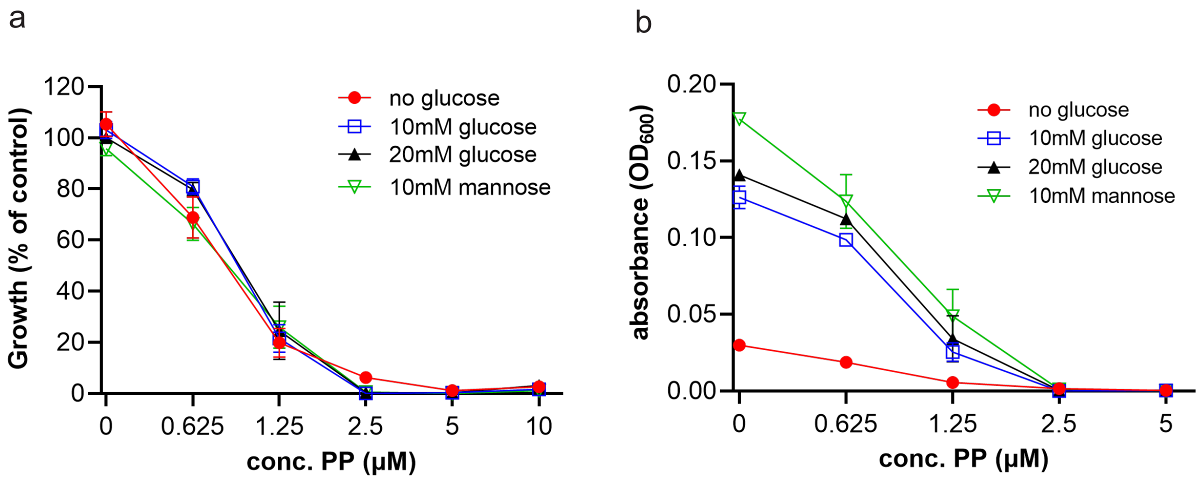

Supplementary Figure 3. Growth inhibition of *C. auris* by pyrvinium pamoate is occurs in a range of glucose concentrations and with mannose as a carbon source. a) *C. auris* was grown in RPMI-1640 medium supplemented with indicated C-source and MIC assays were prepared according to CLSI-M27 procedure. Cell density at 600nm was measured after 20h of growth at 37°C. The data shown are the means ± SEM (n = 3). **b)** Absorbance data used to construct graph in panel a.

Supplementary Figure 4

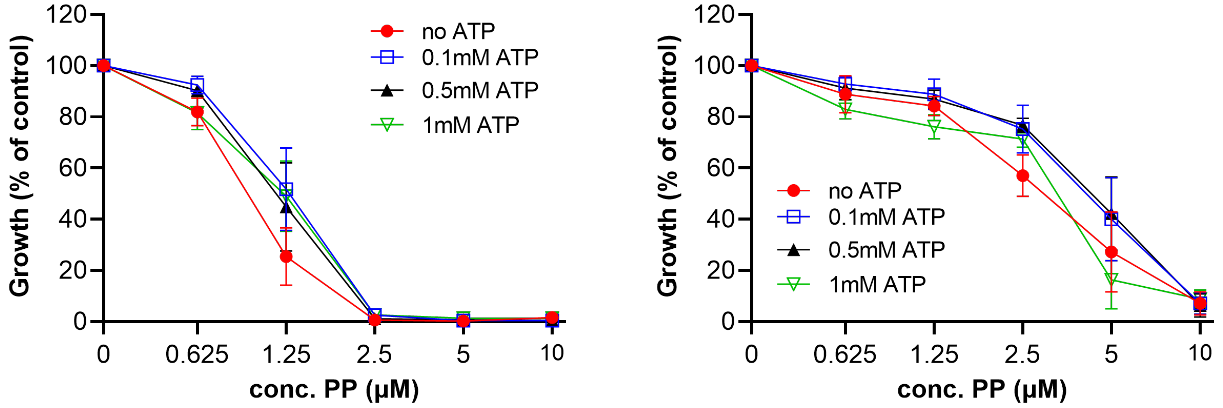

Supplementary Figure 4. ATP supplementation does not rescue *C. auris* from growth inhibitory action of PP *C. auris* was grown in RPMI-1640 medium supplemented with indicated concentrations of ATP and MIC assays were prepared according to CLSI-M27 procedure. Cell density at 600nm was measured after **a)** 20h or **b)** 48h of growth at 37°C. The data shown are the means ± SEM (n = 4).

Supplementary Figure 5

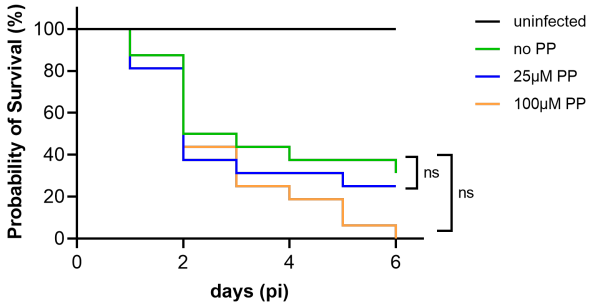

Supplementary Figure 5: Testing the effect of pyrvinium pamoate on the survival of *Galleria mellonella* infected with *C. auris*. Galleria larvae were infected with $5*10^4$ cells of *C. auris* with or without PP treatment. A control group of uninfected larvae was monitored alongside. Survival of larvae was assessed over 6 days. Experiments were done in triplicates with 5 to 6 larvae per treatment group. Kaplan Meyer survival curves of *Galleria mellonella* show the combined data of 16 larvae per treatment group. Statistical differences between the *C. auris* infected treatment groups were calculated by the log-rank Mantel-Cox test (ns $p \geq 0.05$). PP did not show any adverse effect when injected into *Galleria* in the absence of *C. auris* (up to 100 µM, data not shown) and rapid onset of melanisation was observed for all *C. auris* infected larvae suggesting an immediate immune response.

Supplementary Figure 6

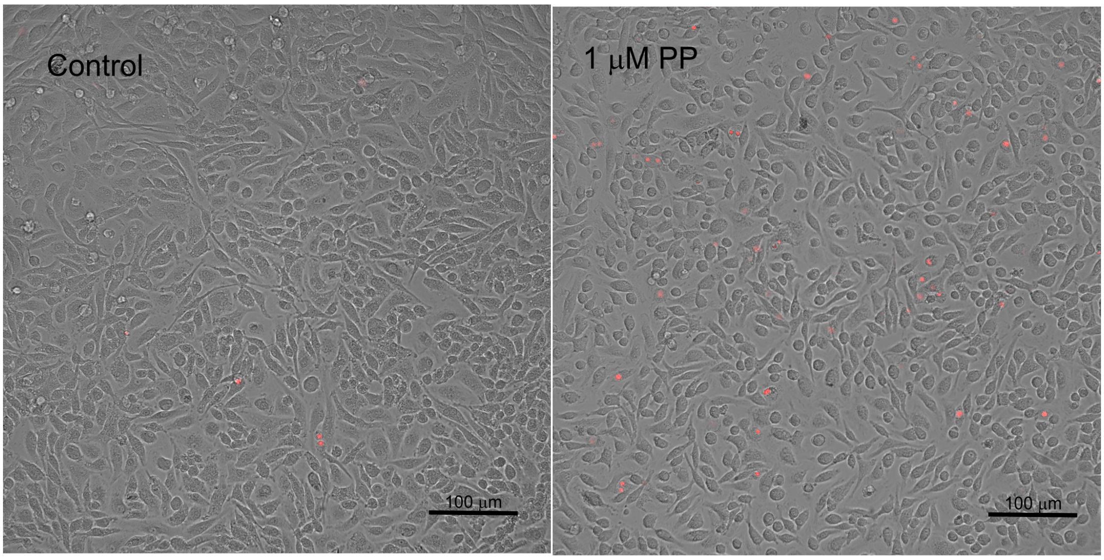

Supplementary Figure 6: Life cell imaging of uninfected BMDMs without and with 1µM PP treatment. DRAQ7 staining of uninfected BMDMs with or without PP treatment at 10h pi at 10x magnification.

Table S1. Strains used in this study.

Strain #	Name	Description	Genotype	Species	Source
YCAT1117	470140	Clinical Isolate, India	Clade 1	*C. auris*	Gift from Sarah Kidd; National Mycology Reference Center, Adelaide.
YCAT1118	470121	Clinical Isolate, India	Clade 1	*C. auris*	Gift from Sarah Kidd; National Mycology Reference Center, Adelaide.
YCAT1191	B11098	Clinical Isolate, Pakistan	Clade 1	*C. auris*	CDC, Atlanta, USA*
YCAT1192	B11109	Clinical Isolate, Pakistan	Clade 1	*C. auris*	CDC, Atlanta, USA
YCAT1193	B11203	Clinical Isolate, India	Clade 1	*C. auris*	CDC, Atlanta, USA
YCAT1194	B11205	Clinical Isolate, India	Clade 1	*C. auris*	CDC, Atlanta, USA
YCAT1195	B11220	Clinical Isolate, Japan	Clade 2	*C. auris*	CDC, Atlanta, USA
YCAT1196	B11221	Clinical Isolate, South Africa	Clade 3	*C. auris*	CDC, Atlanta, USA
YCAT1197	B11222	Clinical Isolate, South Africa	Clade 3	*C. auris*	CDC, Atlanta, USA
YCAT1198	B11244	Clinical Isolate, Venezuela	Clade 4	*C. auris*	CDC, Atlanta, USA
YCAT1199	B11245	Clinical Isolate, Venezuela	Clade 4	*C. auris*	CDC, Atlanta, USA
YCAT1200	B8441	Clinical Isolate, Pakistan	Clade 1	*C. auris*	CDC, Atlanta, USA
YCAT229	SC5314	Clinical isolate, from patient with "*generalised Candida infection*" (Odds et al Genome Biology 2004, 5: 230	Clade 1	*C. albicans*	Traven lab
YCAT607	CBS138	Wildtype strain	Clade 5	*C. glabrata*	ATCC® 2001™
YAT180	W303	Wildtype strain		*S. cerevisiae*	Traven lab

*All isolates from the CDC, Atlanta are held under the material transfer agreement between Centers for Disease Control and Prevention and Monash University D-385-20MTA.
